# Supplementary figures and images for: A firm-level analysis of Chinese commercial health insurance surrender
Source: PLoS One. 2024 Mar 14;19(3):e0296695. doi: 10.1371/journal.pone.0296695 (PMC10939191; doi:10.1371/journal.pone.0296695)

**Log file**

**1、**


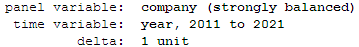


**2、**


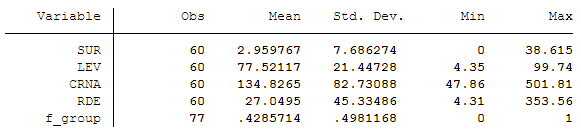


**3、**


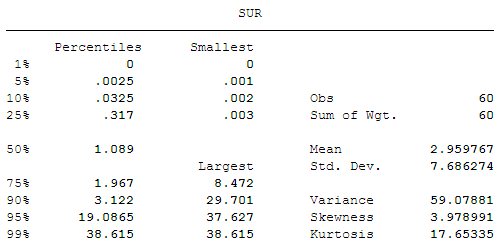


**4、**


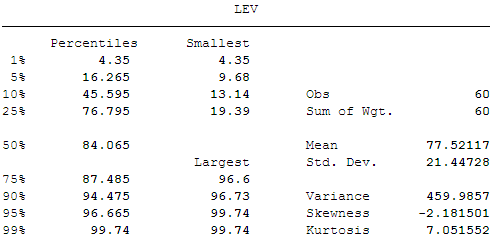


**5、**


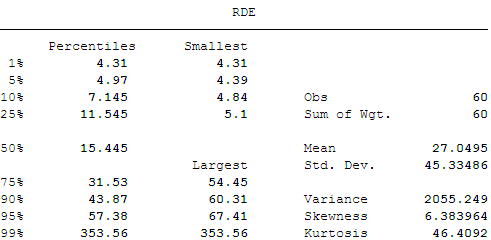


**6、**


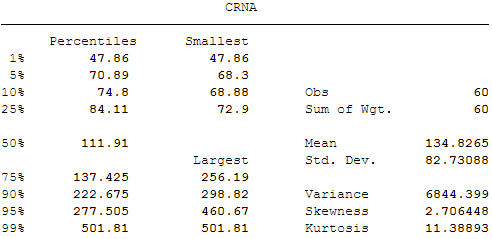


**7、**


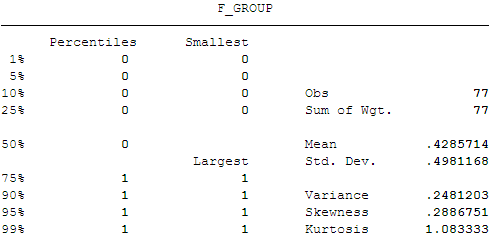


**8、**


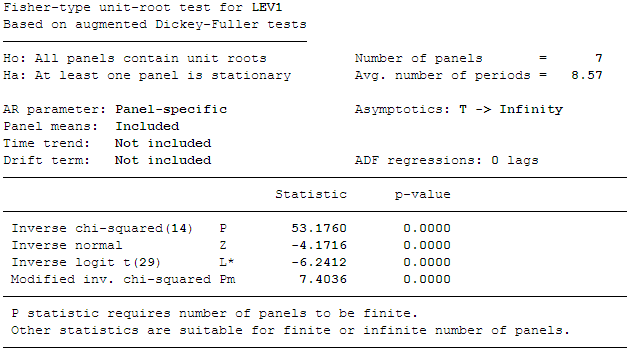


**9、**


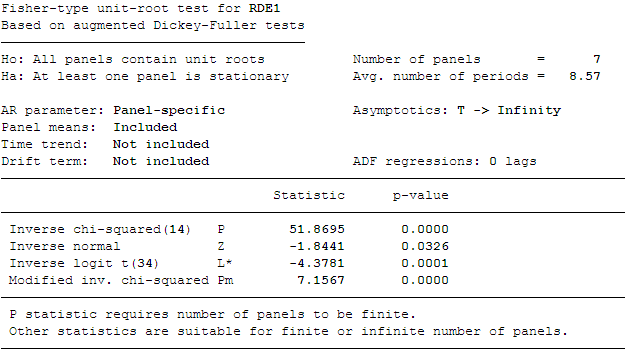


**10、**


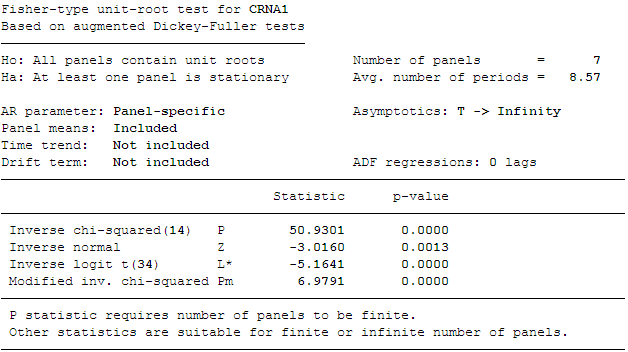


**11、**

**
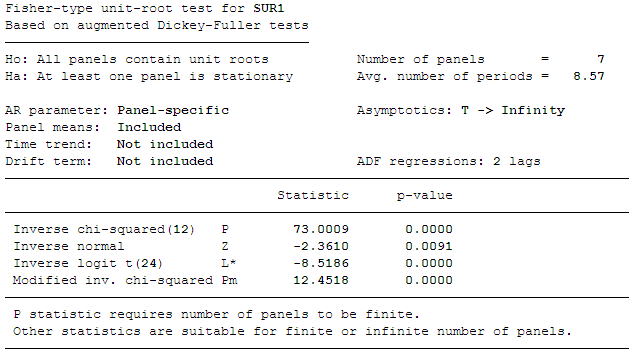
**

**12、**


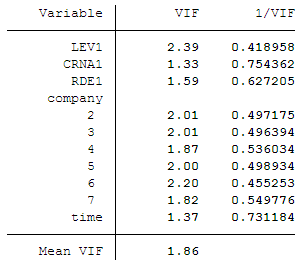


**13、**


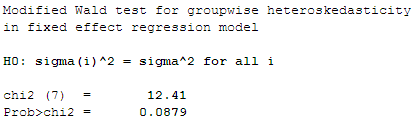


**14、**


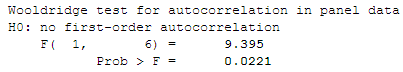


**15、**


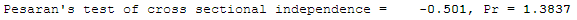


**16、**

**
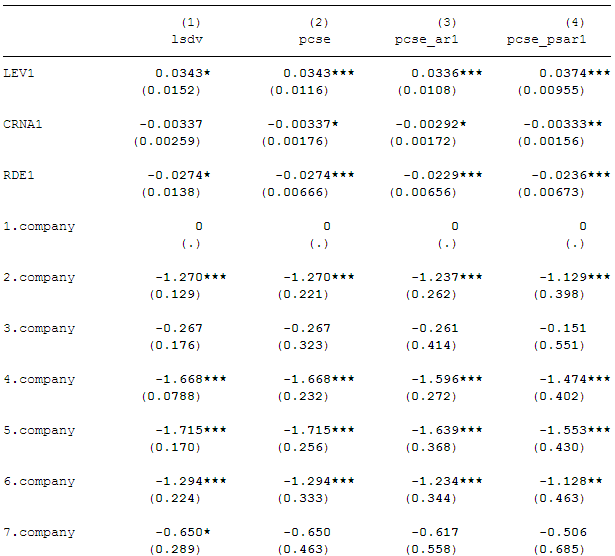
**


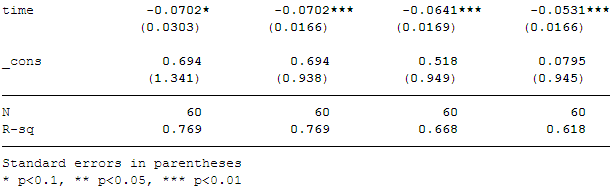


**17、**


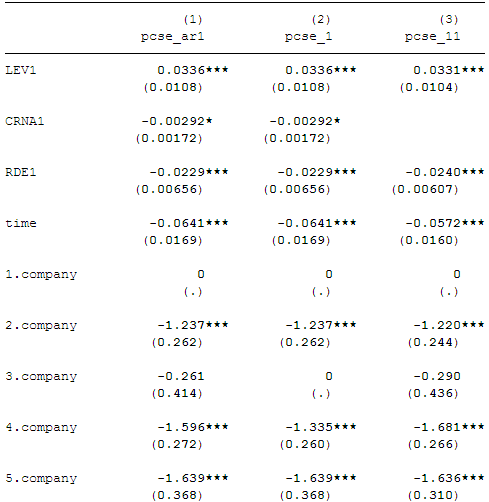


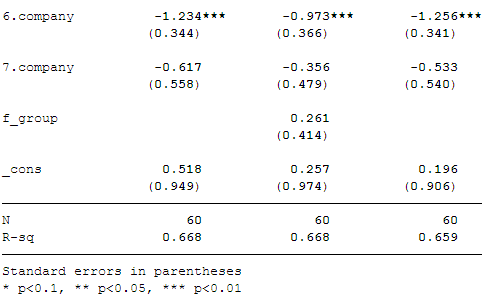

Supplement: S1 File — (DOC) [file pone.0296695.s002.doc]
