# Supplementary material for: A firm-level analysis of Chinese commercial health insurance surrender
Source: PLoS One. 2024 Mar 14;19(3):e0296695. doi: 10.1371/journal.pone.0296695 (PMC10939191; doi:10.1371/journal.pone.0296695)
Supplement: Attachment — Submitted filename: Response to Reviewers.doc [file pone.0296695.s003.doc]

Response to Reviewers

**Reviewer #1:**

1. The authors mention that there are 168 commercial health insurance companies in China as of 2020, but the study only includes data from seven companies. It would be helpful if the authors could provide an explanation for this selection of only seven companies.

**Response：**

**Thank you very much for your comments. Based on your suggestions, we have explained this accordingly. See the red brackets in "1.Introduction" for specific modifications.**

**Thanks again for the reviewer's suggestions.**

2. The introduction section seems to include descriptive statistics, which would be better placed in the empirical analysis section. The introduction should focus on providing background information and addressing the research gap the study aims to fill. Table 1 should be moved to the empirical analysis section, and a condensed version of Section 3 (Theoretical Analysis and Research Hypothesis) should be integrated into the introduction section.

**Response：**

**Thank you very much for your comments. Based on your suggestions, we have expanded and modified "1 Introduction", for details, see the red part of "1.Introduction". However, we choose to leave Table 1 in the "Introduction", because Table 1 is a brief introduction to each professional health insurance company, so that readers can understand the basic information of each professional health insurance company in China more quickly. Therefore, please kindly understand our approach..**

**Thanks again for the reviewer's suggestions.**

3. The statements in lines 192 to 205 appear to be the authors' own conjectures without proper citations. It is essential to support these statements with relevant theoretical and empirical literature.

**Response：**

**Thank you very much for your comments. Based on your suggestion, we have added relevant quotations to lines 192 to 205, mainly adding the literature that asset-liability ratio has a significant positive impact on surrender rate, because there have been empirical studies on this and reached the same conclusion as this paper. In addition, the significant impact of R&D expenditure rate and capital preservation and appreciation rate on surrender rate, It is the extended research finding of this paper to verify the "policy replacement hypothesis" (at the company level), which is the innovation point of this paper, and this part is the theoretical analysis part of our paper. For details, see lines 192 to 205.**

**In addition, we attach the relevant literature cited for reference by the reviewers. Attached:**

[**Yu L**](https://ersp.lib.whu.edu.cn/s/com/webofscience/www/G.https/wos/author/record/18577009)**,** [**Cheng J**](https://ersp.lib.whu.edu.cn/s/com/webofscience/www/G.https/wos/author/record/14772015)**,** [**Lin T T**](https://ersp.lib.whu.edu.cn/s/com/webofscience/www/G.https/wos/author/record/15026739)**. Life Insurance Lapse Behaviour: Evidence from China. Geneva Papers on Risk and Insurance-Issues and Practice. 2019, 44(4):653-678.** [**https://doi.org/10.1057/**](https://doi.org/10.1057/) **s41288-018-0104-5**

**Thanks again for the reviewer's suggestions.**

4. The study design section lacks comprehensive explanations. The selected panel data econometric model, the fixed effect least square dummy variables (LSDV) model, should be described in detail in the study design section. It would be helpful to clarify why this specific model was chosen over other panel data models and provide information on the statistical (econometric) tests to be conducted and the reasoning behind them. Additionally, robustness checks should be discussed in the study design section, rather than in the empirical results section.

**Response：**

**Thank you very much for your comments. First, in accordance with your suggestions, we separate the Robustness test and Endogenous statement from the section on "Empirical Analysis" and make them a separate section, with modifications in "6. Robustness test" and "7. Endogenous description".**

**In addition, we divided three small parts in "4 Research Design", and the third part may cause misunderstanding. Therefore, we changed "4.3 Model setting"to "4.3 Model expression", that is, we modified the part title. Section 4.3 is intended to introduce the model expression. In the model expression, we explain why we are adding a “time trend term” instead of a "time dummy variable" in the usual sense. As for what kind of estimation method is adopted for "long panel data with variable intercept statically unbalanced", through a series of tests in the "5 Empirical Analysis" chapter we concluded that "PW+PCSE" estimation method is the most appropriate estimation method, rather than "LSDV" estimation method.**

**Thanks again for the reviewer's suggestions.**

5. Table 3 should be relocated to the empirical results section for better coherence.

**Response：**

**Thank you very much for your comments. Based on your suggestions, we change "4.2 Data sources and descriptions" to "4.2 Data sources", that is, the title is modified, descriptive statistics of variable data are no longer carried out. Table 3 is repositioned in “5.2 Stability test and descriptive statistics of variable data “”in “5. Empirical Analysis”. For details, see the red section.**

**Thanks again for the reviewer's suggestions.**

6. The results of the various statistical tests (Tables 4, 5, and 6) should be placed in the appendix or supplementary material section, rather than within the main body of the paper.

**Response：**

**Thank you very much for your comments. Since this paper adopts "long panel data with variable intercept statically unbalanced", it is necessary to conduct "multicollinearity test" and "stationarity test". To select a suitable estimation method, therefore, "Test results of inter-group heteroscedasticity, intra-group autocorrelation and inter-group correlation" must be carried out. Therefore, these statistical tests are necessary. At the same time, considering that the length of this paper is not very long, we choose to put them in the body. Therefore, please kindly understand our approach.**

**Thanks again for the reviewer's suggestions.**

**Reviewer #2:**

Comment 1: The introduction is generally fine in it's current state. However, the motivation for this study is completely missing. The contribution of the study is poorly stated and disorganized. It is utterly confusing the purpose of the literature mentioned. The authors should give a clear distinction between this study and existing studies, and provide a clear literature gap.

**Response：**

**Thank you very much for your comments. Based on your suggestions, we have made supplementary revisions to the research contributions in this paper, for details, see the contribution section of "2. Review of Relevant Literature".**

**Thanks again for the reviewer's suggestions.**

Comment 2: The introduction section also did not touch on the main issues in the topic. The authors should conceptualize the terms in the study well and also bring out what other studies have done in this area and what this study seeks to add.

**Response：**

**Thank you very much for your comments. Based on your suggestions, we have expanded and revised the Introduction, for details, see the red part of "1. Introduction".**

**Thanks again for the reviewer's suggestions.**

Comment 3: In the literature review section, the authors should first explain the meaning of commercial health insurance surrender and critically evaluate the literature: Instead of simply summarizing the findings of each study. Discuss any inconsistencies in the existing literature and highlight areas where further research is needed.

**Response：**

**Thank you very much for your comments. Based on your suggestions, we have put the definition and meaning of surrender in the "1. Introduction", highlighted the topic of the article, and revised the "2. Review of Relevant Literature", see the red part of the relevant chapter for specific modifications.**

**Thanks again for the reviewer's suggestions.**

Comment 4: The authors should change the interpretation of results presented in line 327 to 336. Regression and correlation are not the same. Regression shows an effect of one variable on the other and correlation shows linear association.

**Response：**

**Thank you very much for your comments. Based on your suggestions, we have reviewed the relevant data again and believe that the interpretation of the results in lines 327 to 336 is acceptable. The reason is that although regression and correlation are different, they are also related, and correlation analysis is the basis and premise of regression analysis. If there is no correlation, it is meaningless to do regression analysis, that is, regression is based on correlation. Here, we emphasize whether the correlation is positive or negative.**

**In addition, a lot of literature has been expressed in this way, and we attach the relevant literatures for the review of reviewers. The specific literatures are as follows:**

**①Wang Min, He Jie, Xu Peng. Ultimate Control Rights and Corporate Fraud: Evidence from China [J]. Emerging Markets Finance and Trade, 2022, 58(4):1206-1213. DOI:**[**10.1080/1540496X.2020.1845647**](https://scholar.cnki.net/Detail/doi/GARJ2021_2/STJDF9856A6BD714CFDEC092902E610BFB24)**（Page 2）**

**②Ting Shi, Zang Wenbin, Chen Chen, et al. Income distribution and health: What do we know from Chinese data? [J]. PloS one, 2022, 17(1):e0263008-e0263008. DOI:** [**10.1371/JOURNAL.PONE.0263008**](https://scholar.cnki.net/Detail/doi/GARJ2021_2/SJPD813A109B935567CB81D324A608E1B5F8)**（Page 2）**

**③Shen Lu, He Guohua, Yan Huan. Research on the Impact of Technological Finance on Financial Stability: Based on the Perspective of High-Quality Economic Growth [J]. Complexity, 2022. DOI:** [**10.1155/2022/2552520**](https://scholar.cnki.net/Detail/doi/GARJ2021_2/SJHDBDD79A242C5BA081B4CD1EBF2B72BC72)**（Page 3）**

**④Wang Qian, Wang Jun, Gao Feng. Who is more important, parents or children? Economic and environmental factors and health insurance purchase [J]. The North American Journal of Economics and Finance, 2021, 58:101479.DOI:** [**10.1016/J.NAJEF.2021.101479**](https://scholar.cnki.net/Detail/doi/GARJ2021_1/SJESB92E6540E928D688CD7E310E946B7DF9)**（Page 2）**

**However, based on your suggestions, we have made corresponding modifications, specifically, see the red part of "2. Review of Relevant Literature" and the red part of "5.4 Analysis of the empirical results".**

**Thanks again for the reviewer's suggestions.**

Comment 5: The authors should discuss the significance of the coefficients in the regression analysis. It would be useful to provide some economic or practical significance to these findings. Additionally, the authors could compare the findings to existing literature or theoretical expectations to provide a more comprehensive analysis.

**Response：**

**Thank you very much for your comments. Based on your suggestions, we discussed the significance of each coefficient in the regression analysis and compared the research results with existing literatures. For details, see the red part of "5.4 Analysis of the empirical results".**

**Thanks again for the reviewer's suggestions.**

Comment 6: The conclusion of the study is weak, the authors need to indicate the major factors that must be improved to reduce surrender rate in insurance sector in China.

**Response：**

**Thank you very much for your comments. Based on your suggestions, we have given emphasis in the Policy Revelation, as amended in red in "8.2 Policy enlightenments".**

**Thanks again for the reviewer's suggestions.**

**Reviewer #3:**

1* There is the need to offer justification for focusing on health insurance surrender in China. What is the current situation? what is/are the likely implications if the trend remains same? What are the related policy issues that warrant this study.

**Response：**

**Thank you very much for your comments. Based on your suggestions, we have revised the "Introduction", for details, see the red part of "1. Introduction".**

**Thanks again for the reviewer's comments.**

2* The discussion of the results should be improved. For each KEY variable tell the effect it has on "surrender" and offer possible reasons for the outcome in China. Relate the discussion to the literature and previous empirical studies/findings.

**Response：**

**Thank you very much for your comments. Based on your suggestions, we have revised this, and see the red section of "5.4 Analysis of the empirical results" for details.**

**Thanks again for the reviewer's comments.**
