# Supplementary material for: A firm-level analysis of Chinese commercial health insurance surrender
Source: PLoS One. 2024 Mar 14;19(3):e0296695. doi: 10.1371/journal.pone.0296695 (PMC10939191; doi:10.1371/journal.pone.0296695)
Supplement: Attachment — Submitted filename: Response to Reviewers.doc [file pone.0296695.s004.doc]

Response to Reviewers

**Reviewer #1:**

Thank you for your responses to my comments. The subject matter of this article is very important. However, to improve the quality of the paper, I reiterate one of my earlier comments below.

1. The introduction should focus on providing background information and addressing the research gap the study aims to fill. Table 1 should be moved to the empirical analysis section, and a condensed version of Section 3 (Theoretical Analysis and Research Hypothesis) should be integrated into the introduction section.

**Response：**

**Dear Reviewer Kwadwo Arhin:**

**Thank you very much for your comments. Based on your suggestion again, we have revised this, specifically see the red part of "1. Question Raised and Research Hypothesis".**

**Thanks again for your suggestion.**

**Reviewer #2:**

4. Have the authors made all data underlying the findings in their manuscript fully available?

The [PLOS Data policy](http://www.plosone.org/static/policies.action#sharing) requires authors to make all data underlying the findings described in their manuscript fully available without restriction, with rare exception (please refer to the Data Availability Statement in the manuscript PDF file). The data should be provided as part of the manuscript or its supporting information, or deposited to a public repository. For example, in addition to summary statistics, the data points behind means, medians and variance measures should be available. If there are restrictions on publicly sharing data—e.g. participant privacy or use of data from a third party—those must be specified.

**Response：**

**Dear Reviewer Gideon Mensah:**

**Thank you very much for your comments. Based on your suggestion, We have deleted the subdata and provided the master data, as detailed in “Data.xls”.**

**Thanks again for your suggestion.**
